# Pretreatment quality of life as a predictor of survival for patients with nasopharyngeal carcinoma treated with IMRT

**DOI:** 10.1186/s12885-018-4003-8

**Published:** 2018-01-31

**Authors:** Shan-Shan Guo, Wen Hu, Qiu-Yan Chen, Jian-Mei Li, Shi-Heng Zhu, Yan He, Jia-Wen Li, Le Xia, Lu Ji, Cui-Ying Lin, Li-Ting Liu, Lin-Quan Tang, Ling Guo, Hao-Yuan Mo, Chong Zhao, Xiang Guo, Ka-Jia Cao, Chao-Nan Qian, Mu-Sheng Zeng, Ming-Huang Hong, Jian-Yong Shao, Ying Sun, Jun Ma, Yu-Ying Fan, Hai-Qiang Mai

**Affiliations:** 1 State Key Laboratory of Oncology in South China, Collaborative Innovation Center for Cancer Medicine, Sun Yat-Sen University Cancer Center, Guangzhou, 510060 People’s Republic of China; 2 Department of Nasopharyngeal Carcinoma, Sun Yat-Sen University Cancer Center, 651 Dongfeng Road East, Guangzhou, 510060 People’s Republic of China; 3 Good Clinical Practice center, Sun Yat-Sen University Cancer Center, 651 Dongfeng Road East, Guangzhou, 510060 People’s Republic of China; 4 Department of Molecular Diagnostics, Sun Yat-Sen University Cancer Center, 651 Dongfeng Road East, Guangzhou, 510060 People’s Republic of China; 5 Department of Radiation Oncology, Sun Yat-Sen University Cancer Center, Guangzhou, 510060 People’s Republic of China

**Keywords:** Nasopharyngeal carcinoma, Quality of life, EBV DNA, Survival, Prognostic factor

## Abstract

**Background:**

To evaluate the prognostic significance of pretreatment quality of life for patients with nasopharyngeal carcinoma treated with intensity-modulated radiotherapy.

**Methods:**

We performed a prospective, longitudinal study on 554 newly diagnosed patients with NPC from April 2011 to January 2015. A total of 501 consecutive NPC patients were included. Patients were asked to complete the EORTC QLQ-C30 (version 3.0) and QLQ-H&N35 questionnaires before treatment.

**Results:**

Global health status among QLQ-C30 correlates with EBV DNA(*P* = 0.019). In addition, pretreatment appetite loss was significantly correlated with EBV DNA(*P* = 0.02). Pretreatment teeth, opening mouth, feeding tube was significantly correlated with EBV DNA, with *P* value of 0.003, < 0.0001, and 0.031, respectively. In multivariate analysis, pretreatment cognitive functioning of QLQ-C30 was significantly associated with LRFS, with HR of 0.971(95%CI 0.951–0.990), *P* = 0.004. Among scales of QLQ-H&N35 for multivariate analysis, pretreatment teeth (*P* = 0.026) and felt ill (*P* = 0.012) was significantly associated with PFS, with HR of 0.984 (95%CI 0.971–.998) and 1.004 (95%CI 1.001–1.007), respectively. Felt ill of QLQ-H&N35 was significantly associated with DMFS, with HR of 1.004(95%CI 1.000–1.007), *P* = 0.043. There is no QoL scale significantly associated with OS after multivariate analysis.

**Conclusions:**

In conclusion, our analysis confirms that pretreatment teeth and felt ill was significantly associated with PFS in NPC patients treated with IMRT. In addition, the posttreatment EBV DNA was significantly associated with OS.

## Background

Nasopharyngeal carcinoma (NPC) is prevalent in Southern China and Southeast Asia, but rare in the Western world. The annual incidence of NPC is 15–50 cases per 100,000 [1]. NPC differs from other head and neck cancers in its epidemiology, association with Epstein-Barr virus (EBV), and high risk of distant metastasis [2]. Radiotherapy (RT) is the primary treatment for nonmetastatic disease [3, 4]. Intensity modulated radiation therapy (IMRT) is the most frequently recommended radiation method, if conditions permit, because of excellent local control. Concurrent chemoradiotherapy (CCRT) is recommended as a first line therapy for locally advanced NPC [5, 6]. Induction chemotherapy has been combined in several studies to improve clinical outcomes, but it remains controversial [7–9]. Distant metastasis is the major cause of mortality in NPC patients.

Quality of life (QoL) has been considered to be a prognostic factor for cancer patients, such as for head and neck cancer [10, 11], hepatocellular carcinoma and cholangiocarcinoma [12], colorectal cancer [13], liver cancer [14] and lung cancer [15]. Few studies have explored the prognostic significance of pretreatment QoL in NPC [16, 17]. Therefore, we conducted a prospective study using two self-administered questionnaires, the European Oganization for Research and Treatment of Cancer (EORTC) Quality of Life Questionnaire C30 (QLQ-C30) and the EORTC QLQ Head and Neck Cancer–Specific Module (H&N35), to assess the pretreatment QoL scores [18]. We assumed that felt ill among the H&N35 questionaire was significantly associated with PFS.

## Methods

### Patients

We performed a prospective, longitudinal study on 554 newly diagnosed patients with NPC in the Sun Yat-Sen University Cancer Center from April 2011 to January 2015. A total of 501 consecutive NPC patients were included in this study. This study was approved by the clinical research ethics committee of the Sun Yat-Sen University Cancer Center, and the participants provided written informed consent. Patients with the following characteristics were excluded: those with distant metastasis at initial diagnosis (*n* = 10), those lost to follow-up posttreatment (*n* = 2), those whose treatment was interrupted (*n* = 1), those who were unable to complete the questionnaire pretreatment (*n* = 1), those who were unable to complete the questionnaire posttreatment (*n* = 3), those who were unable to complete the questionnaire three months posttreatment (*n* = 3), those who did not test for EAIgA and VCAIgA before treatment (*n* = 10), those who did not test for EBV DNA before treatment (*n* = 17), and those who did not test for EBV DNA value posttreatment (*n* = 7). All patients were given a complete physical examination, a fiber-optic nasopharyngoscopy, magnetic resonance imaging (MRI) of the head and neck, chest radiography, abdominal sonography, electrocardiography, bone scan or PET/CT, complete blood count with a differential count, biochemical profile, and Epstein–Barr virus serology.

### QoL assessments

The self-administered EORTC QLQ-C30 (version 3.0) and the QLQ-H&N35 questionnaires were prospectively given to the enrolled patients [18–20]. The questionnaires are used by a large number of research groups in cancer clinical trials and have also been used in various other, non-trial studies. The Taiwan Chinese version was available and easily completed by our patients. Patients were asked to complete the Chinese version of the EORTC QLQ-C30 (version 3.0) and QLQ-H&N35 questionnaires before treatment. The QLQ-C30 contains 15 scales: five functional scales (physical, role, emotional, cognitive, and social functioning), three symptom scales (fatigue, nausea and vomiting, pain), six single-item symptom scales (dyspnea, insomnia, appetite loss, constipation, diarrhea, financial difficulties), and one global health status/QoL scale. The QLQ-H&N35 is meant for use among head and neck cancer patients with varying disease stages and treatment modalities. The QLQ-H&N35 is composed of seven multi-item symptom scales (pain, swallowing, sensation, speech, eating from a social perspective, social interactions, and sexuality) and 11 single-item symptom scales (teeth, opening mouth, dry mouth, sticky saliva, coughing, felt ill, pain medication use, nutritional supplementation, feeding tube requirement, weight loss, and weight gain). All of the scales and items ranged in score from 0 to 100. A high score for a functional or global QoL scale represents a relatively high/healthy level of functional or global QoL, whereas a high score for a symptom scale or item represents a high number of symptoms or problems.

### Study treatments

#### RT techniques

All patients (501 patients) were treated with IMRT. The dose fractionation and total dose of IMRT for NPC patients followed the guidelines of our institute [21, 22], which are in accordance with the International Commission on Radiation Units and Measurements reports 50 and 62. All the target volumes were depicted slice-by-slice on the treatment planning computed tomography scan. The primary nasopharyngeal gross tumor volume (GTVnx) and the involved cervical lymph nodes were determined based on imaging, clinical, and endoscopic findings. The enlarged retropharyngeal nodes together with primary gross tumor volume (GTV) were outlined as the GTVnx on the IMRT plans. The first clinical tumor volume (CTV1) was defined as the area from 0.5–1.0 cm outside the GTV, a site that involves potential sites of local infiltration. The clinical target volume 2 (CTV2) was defined as the margin from 0.5–1.0 cm around CTV1 and the lymph node draining area (Levels II, III, and IV). For stage N1–3 patients, the lower neck area received conventional anterior cervical field radiation with a midline shield to 50 Gy in daily fractions of 2 Gy. For patients with stage N0 disease, RT was not delivered to the lower neck area. The prescribed dose was 66–70 Gy to the planning target volume (PTV), 60 Gy to PTV1, 54 Gy to PTV2, and 60–66 Gy to the PTV of the involved cervical lymph nodes in 30 to 33 fractions. In total, 30–33 fractions were administered at 1 fraction per day, 5 days per week.

#### Chemotherapy

Patients with clinical stage I were treated with RT alone. Patients with stage II-IVa were treated with CCRT or induction chemotherapy+CCRT. A total of 249 (49.7%) patients received induction chemotherapy followed by CCRT, the regimen of induction chemotherapy regimens were various regimens of based on cisplatin. Overall, 214 (42.7%) patients received concomitant chemotherapy with cisplatin. Of the 214 patients treated with concomitant chemotherapy of cisplatin regimen, a total of 37 patients received cumulative cisplatin dose of < 100 mg/m^2^, 123 patients received cumulative cisplatin dose of 101–200 mg/m^2^ and 54 patients received cumulative cisplatin dose of 200–300 mg/m^2^. A total of 38 patients (7.6%) were treated with RT alone.

### Follow-up and study endpoints

Patients were followed up every 3 months throughout the first 3 years, every 6 months for the next 2 years and annually thereafter. Physical examinations, nasopharyngoscopic examinations, MRIs, chest X-rays, abdominal ultrasounds and EBV DNA tests were performed at each follow-up visit. The follow-up duration was calculated from the first day of treatment to either the day of death or the day of the last examination. The median follow-up duration was 32 months (6–57 months). The primary end point of this study was progression free survival (PFS), and the secondary end points were overall survival (OS), local recurrence-free survival (LRFS) and distant free survival (DMFS). PFS was defined as the time from treatment of NPC to events that included death or disease progression at local, regional, or distant sites or until the date of the last follow-up. OS was defined as the time from treatment of NPC to the date of death or until the date of the last follow-up. LRFS was defined as the time from treatment of NPC to the absence of a primary site or neck lymph node relapse or until the date of the last follow-up. DMFS was defined as the time from treatment of NPC to the date of the first observation of distant metastases or until the date of the last follow-up. The last follow-up date was February 6, 2016.

### Statistical methods

All analyses were performed using SPSS version 18.0 (version 18.0; SPSS Inc., Chicago, III). All tests were 2-tailed. The correlation between EBV DNA and QoL scale was analyzed by Spearman’s correlation .

Univariate analysis measured by the Cox proportional hazards regression model was used to calculate the *P* value of each QoL scale from QLQ-C30 and H&N35. When the *P* value of the QoL scale in univariate analysis was less than 0.05, the scale was separately calculated by multivariate analysis adjusted for age (< 45 vs. ≥ 45), gender (male vs. female), marriage (yes vs. no), education (<high school vs. ≥high school), smoking history (yes vs. no), alcohol history (yes vs. no), T stage (T1,2 vs. T3,4), N stage (N1,2 vs. N3,4), pre-treatment EBV DNA (< 4000 vs. ≥4000) and post-treatment EBV DNA (negative vs. positive).

## Results

### Patient characteristics

In this study population, there were 380 male patients and 121 female patients, with a male: female ratio of 3.14:1. The median age was 44 years (range, 11–72 years). There were 498 (99.4%) of the 501 patients had World Health Organization (WHO) type II or III disease, and 3 (0.6%) had WHO type I disease. There were 9 (1.8%) patients with American Joint of Cancer Committee (AJCC) stage I; 50(10.0%) patients with stage II, 281 (56.1%) patients with stage III, 161 (32.1%) patients with stage IV. A total of 496 (99.0%) patients had an Eastern Cooperative Oncology Group (ECOG) score of 1. More than half of the patients (337, 67.3%) had a history of smoking, and the use of alcohol was not common (53, 10.6%). We represented the characteristics divided by sex in Table 1.

**Table 1:** Patient characteristics (*n*=501)

Variable	Male	Female	*P*
Median age, years			
Range			
< 45	177(46.6%)	75(62.0%)	0.003
≥45	203(53.4%)	46(38.0%)	
Marital status			
Married	17(4.5%)	6(5.0%)	0.833
Single	363(95.3%)	115(95.0%)	
Education years			
No formal education	6(1.6%)	7(5.8%)	0.065
Primary level	49(12.9%)	20(16.5%)	
Secondary level	99(26.1%)	24(19.8%)	
High school	112(29.5%)	37(30.6%)	
University	114(30.0%)	33(27.3%)	
Smoking history			
Ever	159(41.8%)	116(4.1%)	<0.0001
Never	221(58.2%)	5(95.9%)	
Alcohol history			<0.0001
Ever	53(13.9%)	0(0)	
Never	327(86.1%)	121(100.0%)	
ECOG score			0.174
0	378(99.7%)	118(99.2%)	
1	0(0)	1(0.8)	
2	1(0.3%)	0(0)	
WHO type			0.862
1	2(0.5%)	1(0.8%)	
2	2(0.5%)	1(0.8%)	
3	375(98.9%)	117(98.3%)	
T stage			0.521
1	19(5.0%)	3(2.5%)	
2	67(17.6%)	18(14.9%)	
3	202(53.2%)	71(58.7%)	
4	92(24.2%)	29(24.0%)	
N stage			0.641
0	50(13.2%)	11(9.1%)	
1	144(37.9%)	51(42.1%)	
2	146(38.4%)	45(37.2%)	
3	40(10.5%)	14(11.6%)	
AJCC stage			0.322
1	7(1.8%)	2(1.7%)	
2	44(11.6%)	7(5.8%)	
3	207(54.5%)	72(59.5%)	
4	122(32.1%)	40(33.1%)	
Treatment modality			
RT	27(7.1%)	9(7.4%)	
IC + CCRT	194(51.1%)	60(49.6%)	
CCRT	159(41.8%)	52(43.0%)	
Median RT dose, Gy			
VCA IgA			0.188
< 1:80	151(39.7%)	40(33.1%)	
≥ 1:80	229(60.3%)	81(66.9%)	
EA IgA			0.138
< 1:10	199(52.4%)	54(44.6%)	
≥ 1:10	181(47.6%)	67(55.4%)	
Pre-EBV DNA			0.526
≤ 4000	201 (52.9%)	60(49.6%)	
> 4000	179 (47.1%)	61(50.4%)	
Post-EBV DNA			0.780
negative	136(35.8%)	45(37.2%)	
positive	244(64.2%)	76(62.8%)	
Family history of NPC			0.252
yes	18(4.7%)	9(7.4%)	
no	362 (95.3%)	112(92.6%)	

Abbrevations. *No* Number, *ECOG* Eastern Cooperative Oncology Group, *WHO* World Health Organization, *AJCC* American Joint Committee on Cancer, *RT* Radiotherapy, *IC* Induction chemotherapy, *CCRT* Concurrent chemoradiotherapy, *EBV DNA* Epstein-Barr virus deoxyribonucleic acid, *NPC* Nasopharyngeal carcinoma

### Survival outcomes

There were 16 (3.2%) patients who died, 18 (3.6%) patients who had loco regional recurrence and 42 (8.4%) patients who had distant metastasis. The median follow-up time was 32 months (range, 6–57).

### QoL data

Table 2 shows the pretreatment QoL scores of both QLQ-C30 and QLQ-H&N35 for NPC patients.

**Table 2:** Pretreatment quality of life scores for 501 patients with nasopharyngeal carcinoma

EORTC scale	Mean	SD
QLQ-C30		
Global health status/QoL	69.84	22.47
Physical functioning	94.07	9.22
Role functioning	93.88	14.73
Emotional functioning	84.21	16.56
Cognitive functioning	88.39	16.02
Social functioning	75.82	25.88
Fatigue	17.25	17.27
Nausea and vomiting	3.46	10.39
Pain	12.28	18.43
Dyspnoea	6.72	15.10
Insomnia	15.77	23.52
Appetite loss	7.12	16.74
Constipation	6.65	15.78
Diarrhea	3.53	10.49
Financial difficulties	31.27	31.45
QLQ-H&N35		
Pain	8.13	11.20
Swallowing	3.71	9.16
Senses	5.76	12.72
Speech	5.26	12.72
Social eating	4.14	9.39
Social contact	3.97	8.75
Sexuality	16.43	20.42
Teeth	15.90	21.45
Opening mouth	6.59	16.16
Dry mouth	17.22	19.72
Sticky saliva	11.50	17.96
Coughing	9.51	17.14
Felt ill	24.42	26.59
Pain killers	20.96	40.74
Nutrition supplements	20.96	40.74
Feeding tube	1.20	10.89
Weight loss	34.53	47.59
Weight gain	7.19	25.85

Abbrevations. *EORTC* European Organisation for Research and Treatment of Cancer, *SD* Standard deviation

### Correlation between EBV DNA and QoL

We analyzed correlation between each scale among the QLQ-C30 questionnaire and pretreatment EBV DNA, found that global health status correlates with pretreatment EBV DNA(*P* = 0.019). In addition, pretreatment appetite loss was significantly correlated with pretreatment EBV DNA(*P* = 0.02). We also analyzed the correlation between each scale among the QLQ-H&N35 questionnaire and pretreatment EBV DNA. We found that pretreatment teeth, opening mouth, feeding tube was significantly correlated with pretreatment EBV DNA, with *P* value of 0.003, < 0.0001, and 0.031, respectively. Appendix: Tables 7 and 8 represented the correlation between EBV DNA and QLQ-C30 or QLQ-H&N35.

### Univariate analysis pretreatment

**Fig. 1:**
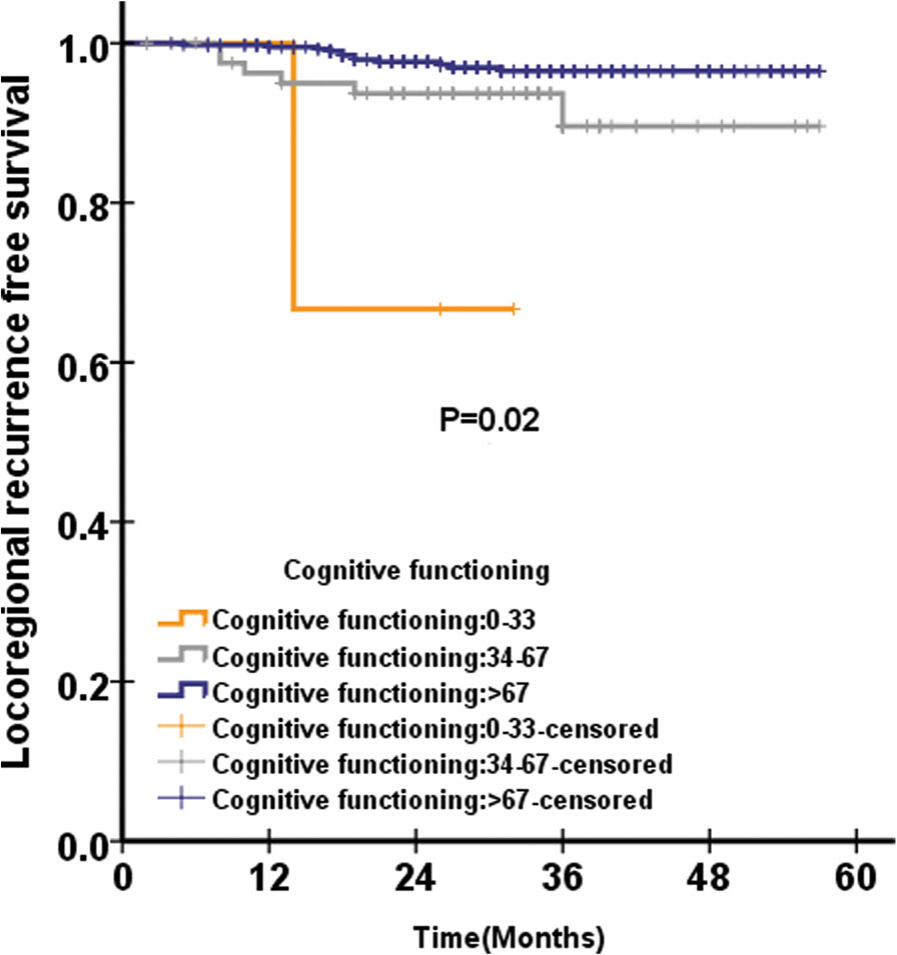
Distant metastasis free survival according to pretreatment felt ill score of QLQ-H&N35 questionnaire among 501 patients with NPC analysed by Kaplan-Meire and log-rank method

In QLQ-C30, there was no functional scale or symptom scale that was significantly associated with OS, PFS and DMFS in QLQ-C30 pretreatment. Only pretreatment cognitive functioning was significantly associated with LRFS in QLQ-C30 (Fig. 1).

**Fig. 2:**
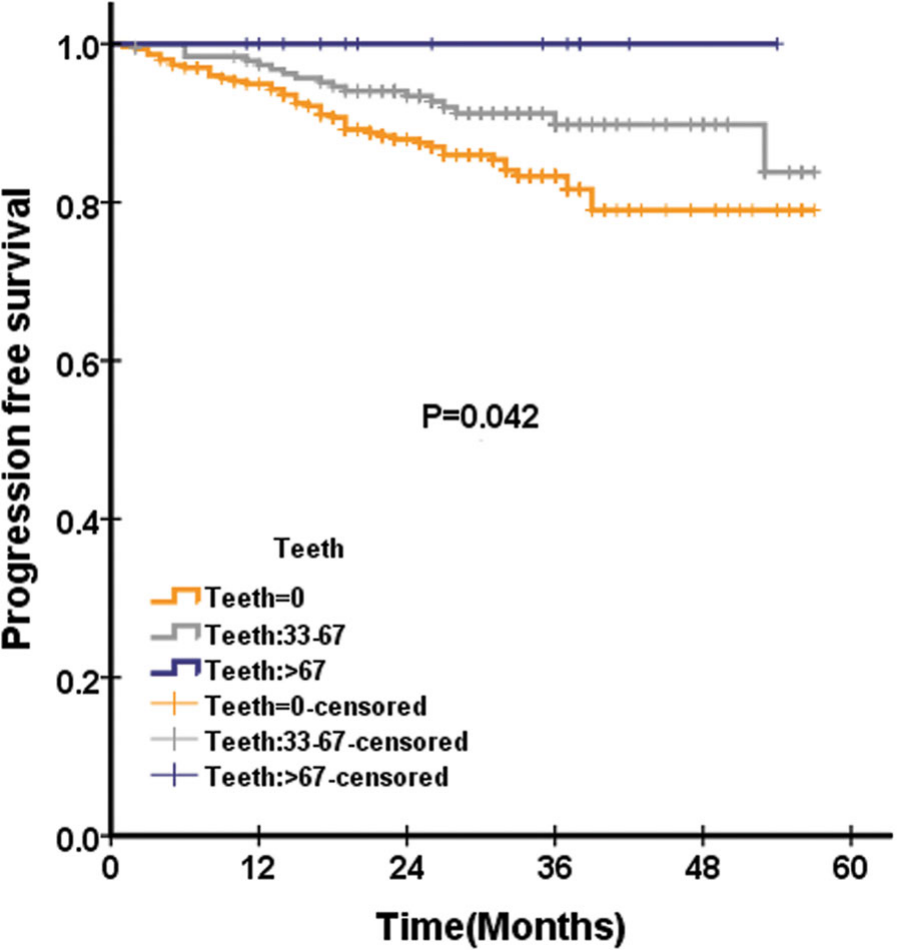
Loco regional recurrence free survival according to pretreatment cognitive functioning score of QLQ-C30 questionnaire among 501 patients with NPC analysed by Kaplan-Meire and log-rank method

**Fig. 3:**
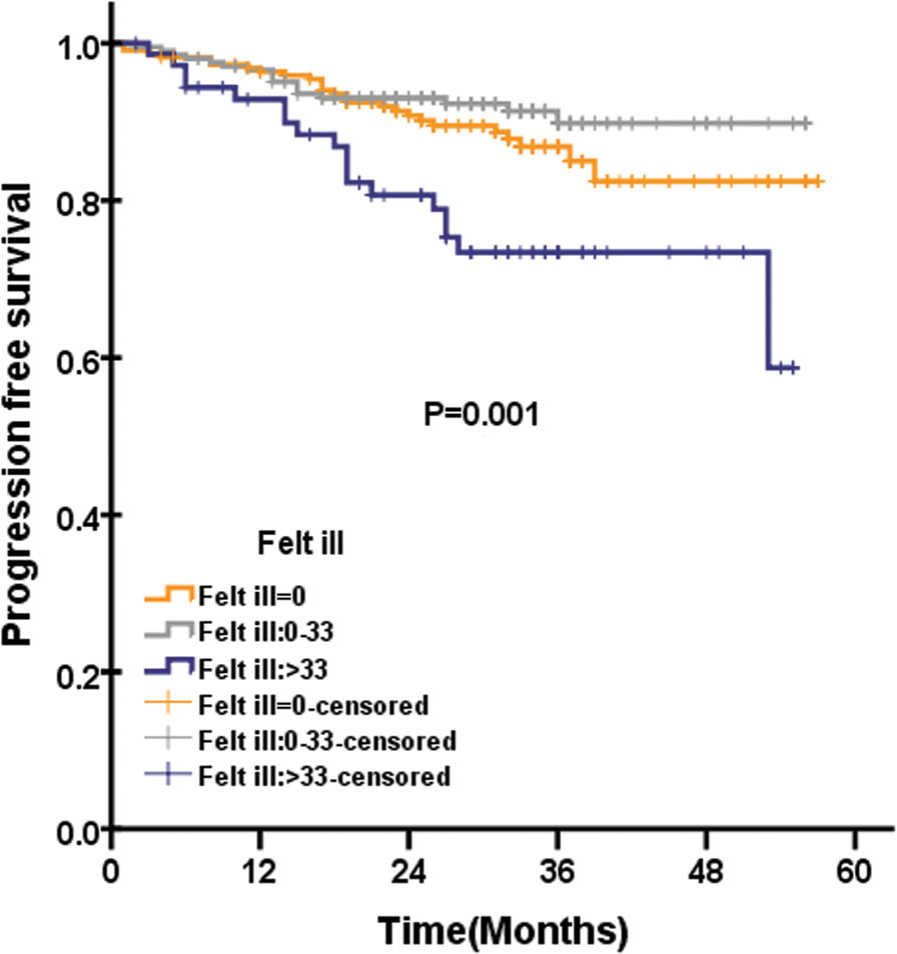
Progression free survival according to pretreatment teeth score of QLQ-H&N35 questionnaire among 501 patients with NPC analysed by Kaplan-Meire and log-rank method

**Fig. 4:**
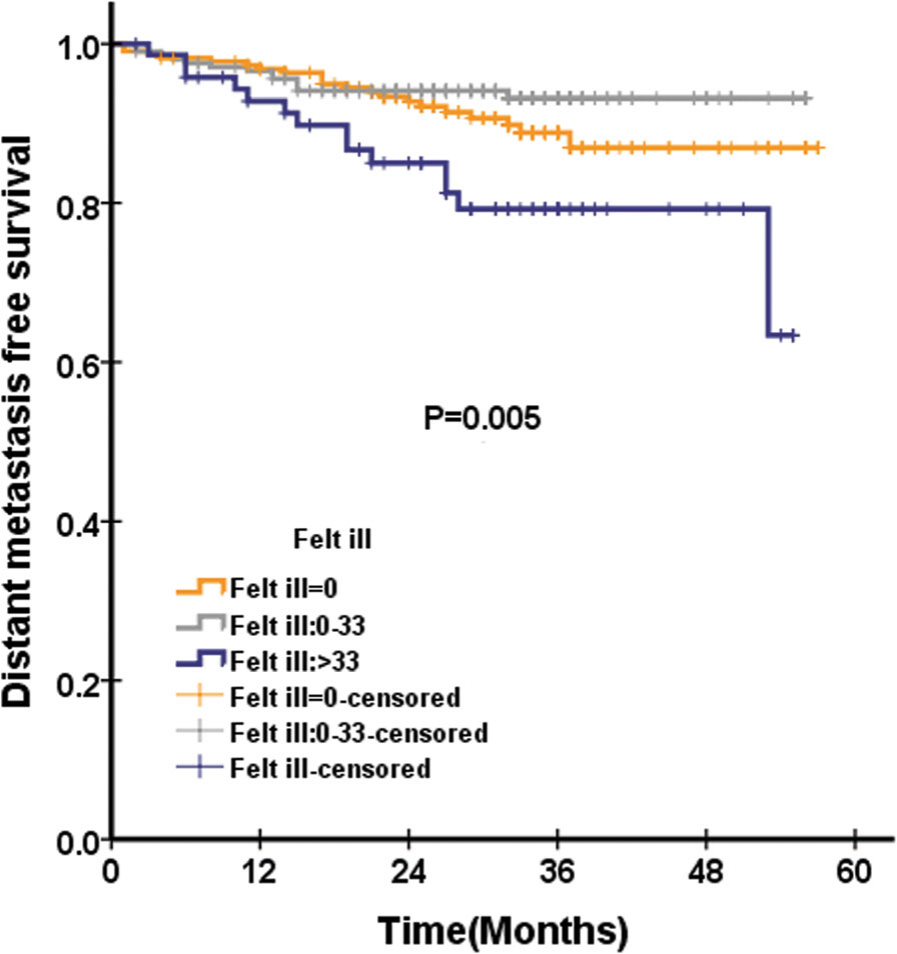
Progression free survival according to pretreatment felt ill score of QLQ-H&N35 questionnaire among 501 patients with NPC analysed by Kaplan-Meire and log-rank method

In QLQ-H&N35, were pain and swallowing significantly associated with OS. There were three scales significantly associated with PFS: pain, teeth (Fig. 2) and felt ill (Fig. 3). There were six scales in QLQ-H&N35 that were significantly associated with LRFS: pain, swallowing, speech, social eating and teeth. There were two scales in QLQ-H&N35 that were significantly associated with DMFS: pain and felt ill (Fig. 4). (Appendix: Table 7).

### Multivariate analysis

The scales which were significantly associated with clinical outcomes were included in Cox proportional hazards regression model (Tables 3, 4, 5 and 6). In multivariate analysis, pretreatment cognitive functioning of QLQ-C30 was significantly associated with LRFS, with HR of 0.971 (95%CI 0.951–0.990), *P* = 0.004. Among scales of QLQ-H&N35 for multivariate analysis, pretreatment teeth (*P* = 0.026) and felt ill (*P* = 0.012) was significantly associated with PFS, with HR of 0.984 (95%CI 0.971–0.998) and 1.004 (95%CI 1.001–1.007), respectively. Besides, posttreatment EBV DNA (*P* = 0.001) and N stage (*P* = 0.013) was significantly associated with PFS, with HR of 3.130 (95%CI 1.563–6.267) and 1.979 (95%CI 1.156–3.388), respectively. Felt ill of QLQ-H&N35 was significantly associated with DMFS, with HR of 1.004 (95%CI 1.000–1.007), *P* = 0.043. Besides, post-treatment EBV DNA (*P* = 0.007) and N stage (*P* = 0.010) was significantly associated with DMFS, with HR of 2.915 (95%CI 1.338–6.350) and 2.251 (95%CI 1.212–4.179). There is no QoL scale significantly associated with OS after multivariate analysis. In addition, the posttreatment EBV DNA was significantly associated with OS (*P* = 0.020), with HR of 11.202 (95%CI 1.473–85.184).

**Table 3:** Multivariate analysis of PFS on pretreatment quality of life of QLQ-C30 among 501 patients with nasopharyngeal carcinoma

	HR	95%CI	*P*
Age	1.200	0.692–2.079	0.516
Gender	0.772	0.395–1.510	0.450
Marriage	1.470	0.458–4.719	0.517
Education	1.197	0.940–1.525	0.145
Smoking history	0.951	0.518–1.747	0.872
Alcohol history	0.787	0.324–1.910	0.596
T stage	1.383	0.684–2.794	0.367
N stage	1.979	1.156–3.388	0.013
Pre-treatment EBV DNA	1.451	0.866–2.431	0.157
Post-treatment EBV DNA	3.130	1.563–6.267	0.001
Pain	1.015	0.995–1.035	0.146
Teeth	0.984	0.971–0.998	0.026
Felt ill	1.004	1.001–1.007	0.012

Abbreviations: *PFS* Progression free survival, *HR* Harsard ratio

**Table 4:** Multivariate analysis of LRFS on quality of life of QLQ-C30 among 501 patients with nasopharyngeal carcinoma

	HR	95%CI	*P*
Age	1.583	0.582–4.302	0.368
Gender	1.017	0.300–3.450	0.978
Marriage	0.479	0.099–2.320	0.360
Education	1.337	0.852–2.098	0.206
Smoking history	1.455	0.512–4.139	0.482
Alcohol history	1.086	0.480–2.458	0.843
T stage	1.267	0.361–4.449	0.712
N stage	1.604	0.616–4.172	0.333
Pre-treatment EBV DNA	1.221	0.491–3.035	0.667
Post-treatment EBV DNA	3.093	0.881–10.857	0.078
Cognitive functioning	0.971	0.951–0.990	0.004

Abbreviations: *LRFS* Loco regional recurrent free survival, *HR* Harsard ratio

**Table 5:** Multivariate analysis of DMFS on pretreatment quality of life of QLQ-C30 among 501 patients with nasopharyngeal carcinoma

	HR	95%CI	*P*
Age	1.039	0.560–1.927	0.904
Gender	0.527	0.235–1.183	0.121
Marriage	3.217	0.669–15.479	0.145
Education	1.278	0.967–1.689	0.085
Smoking history	0.731	0.362–1.477	0.383
Alcohol history	0.882	0.342–2.271	0.794
T stage	1.305	0.611–2.787	0.491
N stage	2.251	1.212–4.179	0.010
Pre-treatment EBV DNA	1.730	0.963–3.109	0.067
Post-treatment EBV DNA	2.915	1.338–6.350	0.007
Pain	1.015	0.994–1.038	0.169
Felt ill	1.004	1.000–1.007	0.043

Abbreviations: *DMFS* Distant metastasis free suvival, *HR* Harsard ratio

**Table 6:** Multivariate analysis of OS on pretreatment quality of life of QLQ-C30 among 501 patients with nasopharyngeal carcinoma

	HR	95%CI	*P*
Age	1.329	0.516–3.427	0.556
Gender	0.493	0.136–1.795	0.284
Marriage	1.524	0.226–10.298	0.665
Education	1.145	0.763–1.720	0.513
Smoking history	1.246	0.483–3.216	0.650
Alcohol history	0.268	0.034–2.125	0.213
T stage	2.353	0.539–10.275	0.255
N stage	1.675	0.686–4.090	0.257
Pre-treatment EBV DNA	0.816	0.342–1.946	0.646
Post-treatment EBV DNA	11.202	1.473–85.184	0.020
Pain	1.028	0.996–1.061	0.091
Swallowing	1.014	0.978–1.050	0.458

Abbreviations: *OS* Overall sruvival, *HR* Harsard ratio

## Discussion

There have been previous studies regarding quality of life on NPC patients and head and neck cancer. Until now, only one study had explored the prognostic significance of QoL in QLQ-C30 questionnaires by assessing 254 NPC patients who received IMRT and 93 patients who received 3DCRT [17]. To our knowledge, this is the first large scale study of NPC patients in the IMRT era that prospectively explored functional scales and symptom scales in both QLQ-30 and H&N35.

We found that global health status significantly correlates with EBV DNA. High pretreatment EBV DNA level always associates with large tumor or multiple lymph nodes which represents advanced stage. Patients with advanced stage represents poor quality of life scores. This may be the possible explanation for global health status significantly correlates with EBV DNA. In addition, pretreatment appetite loss was significantly correlated with EBV DNA. We found that pretreatment teeth, opening mouth, feeding tube was significantly correlated with EBV DNA. This is the first time that the correlation between quality of life and EBV DNA is reported. The exact mechanism remains unknown. More studies about the correlation between quality of life and EBV DNA is expected to do in the future.

In the present study, pretreatment teeth in QLQ-H&N35 predicted longer PFS. This result may be explained by a sensitivity to radiotherapy resulting in uncomfortable sensation in the teeth. The exact mechanism is unknown. Interestingly, felt ill pretreatment in QLQ-H&N35 predicted shorter DMFS in multivariate analysis. The possible explanation would be as follows. At the beginning of treatment, pain mostly comes from large tumor region, probably because of invasion along the cranial nerve. Large tumors of head and neck cancers or NPC are significantly associated with distant metastasis. A previous study found that pretreatment pain influences OS in 2340 newly diagnosed patients with head and neck squamous cancer [23]. We found that a high cognitive functioning score pretreatment in QLQ-C30 predicted longer LRFS. This finding is consistent with previous studies in head and neck cancer [24] and NPC [17]. The exact mechanism of why cognitive function correlates with survival is unknown. The causative relationship between cognitive functioning and survival is indeterminate. Cognitive functioning might be a surrogate for the QoL scales that were potentially prognostic, and we speculate that it may display as a physiological appearance for some undetected predictive factors.

In this study, post treatment EBV DNA predicted OS better than pretreatment EBV DNA. Using multivariate analysis, posttreatment EBV DNA significantly predicted OS for NPC patients in this study. Pretreatment EBV DNA did not show predict value of OS in this study in multivariate analysis, revealed that the prognostic value of pretreatment EBV DNA was covered up by posttreatment EBV DNA in this study. This finding is consistent with previous studies. A recent study explored EBV DNA loading of 273 NPC patients at different time points and found that post treatment EBV DNA was significantly associated with PFS, DMFS and OS [25]. Several studies in Taiwan concluded that post treatment EBV DNA was an important independent prognostic factor for clinical outcomes [26, 27].

Our results revealed that QoL and post treatment EBV DNA can effectively predict survival for NPC patients. The results provide a promising way to guide treatment strategy for NPC patients. Our study has several strengths. First, the present study has the longest longitudinal collection of QoL data that has been used to examine prognostic value during the initial management of patients with NPC. Second, this is the first time that QoL scores in QLQ-H&N35 were found to predict survival for NPC patients. Our study evaluated the prognostic significance of QoL using both the QLQ-C30 questionnaire and QLQ-H&N35 questionnaire.

There were some limitations in the present study. First, this is a single center study in a high incidence area in Southern China. Future studies are needed to calculate the prognostic significance of QoL in NPC patients in other areas in the world. Second, the median follow-up time of this study was 32 months; a longer follow-up time is needed to further validate our results.

## Conclusions

In conclusion, our analysis confirms that pretreatment teeth and felt ill was significantly associated with PFS in NPC patients treated with IMRT. In addition, the posttreatment EBV DNA was significantly associated with OS.

## Appendix

**Table 7:** The correlation between quality of life scales among EORTC QLQ-C30 and EBV DNA

Scale	Β	*P*
Global health status/QoL	0.105	0.019
Physical functioning	0.001	0.983
Role functioning	0.082	0.066
Emotional functioning	0.081	0.071
Cognitive functioning	0.059	0.184
Social functioning	0.056	0.208
Fatigue	−0.080	0.071
Nausea and vomiting	−0.057	0.203
Pain	−0.048	0.280
Dyspnoea	−0.080	0.074
Insomnia	− 0.081	0.069
Appetite loss	−0.104	0.020
Constipation	−0.042	0.351
Diarrhea	−0.059	0.184
Financial difficulties	−0.057	0.203

Abbrevations. *EORTC* European Organisation for Research and Treatment of Cancer, *QoL* Qualtiy of life

**Table 8:** The correlation between quality of life scales among EORTC H&N35 and EBV DNA

Scale	Β	*P*
Pain	− 0.060	0.178
Swallowing	−0.052	0.244
Senses	−0.038	0.397
Speech	−0.042	0.343
Social eating	−0.031	0.488
Social contact	−0.003	0.950
Sexuality	−0.042	0.343
Teeth	−0.134	0.003
Opening mouth	−0.156	< 0.0001
Dry mouth	−0.025	0.583
Sticky saliva	−0.076	0.089
Coughing	−0.042	0.351
Felt ill	−0.030	0.497
Pain killers	−0.065	0.147
Nutrition supplements	−0.068	0.126
Feeding tube	0.096	0.031
Weight loss	−0.007	0.880
Weight gain	0.015	0.739

Abbrevations. *EORTC* European Organisation for Research and Treatment of Cancer, *QoL* Qualtiy of life

## Abbreviations

CCRT: Concurrent chemoradiotherapy
DMFS: Distant free survival
EBV: Epstein-Barr virus
EORTC: European Organization for Research and Treatment of Cancer
H&N35: Head and Neck Cancer–Specific Module
IMRT: Intensity modulated radiation therapy
LRFS: Local regional recurrence-free survival
MRI: Magnetic resonance imaging
NPC: Nasopharyngeal carcinoma
OS: Overall survival
PFS: Progression free survival
QLQ-C30: Quality of Life Questionnaire C30
QoL: Quality of life
RT: Radiotherapy
